# Geometric analysis of soft thresholds in action potential initiation and the consequences for understanding phase response curves and model tuning

**DOI:** 10.1186/1471-2202-13-S1-P165

**Published:** 2012-07-16

**Authors:** Robert Clewley, Bryce Chung

**Affiliations:** 1 Neuroscience Institute, Georgia State University, Atlanta, GA 30303, USA; 2 Department of Mathematics and Statistics, Georgia State University, Atlanta, GA 30303, USA

## 

Recent mathematical and computational work on the space-clamped Hodgkin-Huxley (H-H) model of neural excitability identifies major dynamic features of the changing nullclines in 2D phase plane projections during action potential (AP) initiation [1,2]. Such analysis provides novel mechanistic and geometric understanding (in terms of interplay between variables and currents) of the “soft threshold” dynamics. We perform visualization of the nullcline dynamics around threshold, and characterize important geometric properties via “dominant scale analysis” [3], which avoids the need to make asymptotic approximations. In particular, we analyze the transient dynamics during the passage through the ghost of a saddle-node bifurcation in the (*V*, *m*) phase-plane projection of a local 3D approximation to the 4D H-H equations (where sodium inactivation *h* is held constant). Linear analysis of the moving *V*-nullcline in these projections indicates that a necessary condition for AP initiation is the eventual holding of the following inequalities in the neighborhood of the ghost of the saddle node:

where the asymptotic steady state voltage *V_∞_*(*m*, *n*) is for constant *h*. Given that  and  in this neighborhood, the first inequality can be interpreted as saying that the fast sodium current must dominate the effect of the growing delayed rectifier potassium current, but not in such a way that  becomes large too rapidly. We find that the curvature of the nullclines in this region is responsible for the truth of this condition (for standard parameter values used for Type I and II modes of H-H excitability).

We compare different parameter regimes for periodic and transient trajectories using our analysis. Under mild variation of parameters and initial conditions, and except in pathological circumstances that are related to the generation of sub-threshold oscillations and canards (e.g., see ref. [2]), we can predict the onset of AP initiation and its timing as the result of parameter changes or small voltage perturbations. This leads to insight about the origin of phase response curve shape in Type I vs. Type II neural excitability. Geometric features of the nullclines are measured during this analysis using PyDSTool [4], and the curvature conditions can be used to guide objective function choice for parameter tuning tasks where AP generation is found to be incomplete or imperfect (e.g., compare refs. [5,6]). This has applications in situations where APs are pathologically changed due to genetic channel defects (especially in potassium channels), or where unwanted depolarization block occurs.
